# Vestibular Evoked Myogenic Potential (VEMP) Testing for Diagnosis of Superior Semicircular Canal Dehiscence

**DOI:** 10.3389/fneur.2020.00695

**Published:** 2020-07-21

**Authors:** Kimberley S. Noij, Steven D. Rauch

**Affiliations:** ^1^Department of Otolaryngology, Massachusetts Eye and Ear, Boston, MA, United States; ^2^Department of Otolaryngology, Harvard Medical School, Boston, MA, United States

**Keywords:** third window syndrome, semicircular canal dehiscence syndrome, vestibular evoked myogenic potential, diagnostic, otology

## Abstract

Superior semicircular canal dehiscence is a bony defect of the superior semicircular canal, which can lead to a variety of auditory and vestibular symptoms. The diagnosis of superior semicircular canal dehiscence (SCD) can be challenging, time consuming, and costly. The clinical presentation of SCD patients resembles that of other otologic disease, necessitating objective diagnostics. Although temporal bone CT imaging provides excellent sensitivity for SCD detection, it lacks specificity. Because the treatment of SCD is surgical, it is crucial to use a highly specific test to confirm the diagnosis and avoid false positives and subsequent unnecessary surgery. This review provides an update on recent improvements in vestibular evoked myogenic potential (VEMP) testing for SCD diagnosis. Combining audiometric and conventional cervical VEMP results improves SCD diagnostic accuracy. High frequency VEMP testing is superior to all other methods described to date. It is highly specific for the detection of SCD and may be used to guide decision-making regarding the need for subsequent CT imaging. This algorithmic sequential use of testing can substantially reduce radiation exposure as well as cost associated with SCD diagnosis.

## Introduction

Superior semicircular canal dehiscence is a bony defect of the superior semicircular canal (SSC), which can lead to a variety of symptoms, including sound and pressure induced dizziness, aural fullness, hearing loss, autophony, hyperacusis, and pulsatile tinnitus (1). These symptoms are thought to occur due to a “Third Window” mechanism caused by the dehiscence. In the presence of normal bony covering of the semicircular canals, sound stimulation of the ear causes the stapes footplate and oval window to move, resulting in a pressure wave across the basilar membrane in the cochlea and an equal outward motion of the round window. In the presence of a dehiscence, the energy created by stapes footplate and oval window motion is shunted away from its usual route and toward the third window. As a result, the pressure difference across the basilar membrane in the cochlea decreases and energy transmission to the vestibular sense organs increases (2, 3).

In the early twentieth century, Tullio et al. described that fenestration of the semicircular canals in pigeons led to sound-induced eye and head motion in the plane of the fenestrated canal, indicating activation of the vestibulo-ocular and vestibulocollic pathways (4–6). In 1998, Minor et al. were the first to describe this combination of the anatomical defect and symptoms in humans, dubbed superior semicircular canal syndrome (SCDS). Treatment of SCDS is reserved for patients with disabling or severely intrusive symptoms and consists of surgical plugging of the dehiscence (1).

Many auditory and vestibular symptoms experienced by SCDS patients also occur in other otologic pathologies, such as otosclerosis and Meniere's disease. SCDS patients have even undergone unsuccessful surgical procedures, such as stapedectomies, before being correctly diagnosed (7). It is therefore essential to use objective diagnostics to differentiate SCDS from other pathologies and to confirm diagnosis.

Because there is no single gold standard definitive diagnostic test for SCDS, its diagnosis is generally based on a combination of symptomatology, threshold audiometry, and immittance testing, video-oculography, temporal bone CT imaging, and vestibular evoked myogenic potential (VEMP) testing (3, 8). The choice of diagnostic tools is dependent on their availability and therefore varies per institution. Thus, SCDS diagnosis is often not straightforward, it can be time consuming, and it can be costly.

## Diagnostic Tools and Challenges

The initial cohort of SCDS patients described by Minor et al. suffered from sound- and/or pressure-induced vestibular symptoms (1). Eye movements in the plane of the superior semicircular canal were observed with video-oculography or magnetic field search-coil recordings in 7 of 8 patients. Patients underwent temporal bone CT imaging in the axial and coronal planes (1 mm slice thickness) and all showed a dehiscence of the superior semicircular canal. Brain MRI with and without IV gadolinium performed in 6 patients were normal (1).

Over time, the diagnostic approaches to SCDS patients have been refined and SCDS diagnosis is currently based on a test battery approach (3, 8). Since symptoms that give rise to consideration of the SCDS diagnosis can be auditory, vestibular or both, both auditory and vestibular testing, as well as imaging, play important roles.

## Temporal Bone CT Imaging

**Figure 1:**
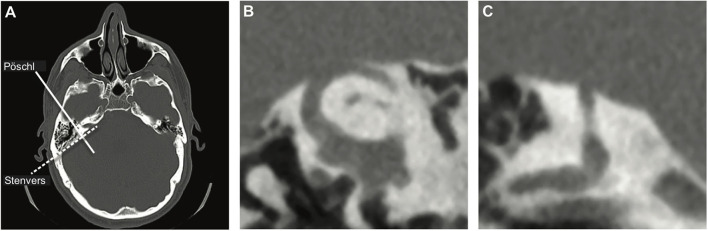
CT images. **(A)** Axial view of the head indicating reformatting planes parallel (Pöschl—solid line) and perpendicular (Stenvers—dashed line) to the plane of the superior semicircular canal (SSC). **(B)** Pöschl view and **(C)** Stenvers view of a dehiscent SSC. The normal bony covering of the SSC is clearly absent in both views.

Since the issue in SCDS constitutes an anatomical defect, obtaining imaging of the temporal bone to assess the SSC seems a logical diagnostic choice and is widely used to assess patients suspected of SCD, although relatively costly (1). Ideally, CT images are evaluated in the planes parallel (Pöschl) and perpendicular (Stenvers) to the plane of the SSC (Figure 1). This diagnostic modality is highly sensitive but lacks specificity; i.e., it is highly likely to detect any true dehiscence but may also give rise to false positives, suggesting dehiscence when none is there. Clinical CT scans overestimate both the presence and size of the dehiscence, especially when the layer of bone covering the canal is thin and when only the Stenvers view is used (9–11). Theoretically, the use of a finer slice thickness would improve the specificity of CT imaging but that is accompanied by an increased risk of motion artifact and increased radiation exposure. Because the treatment of SCDS is surgical, it is crucial to use a highly specific test to confirm the diagnosis and avoid false positives.

## Audiometry

As SCDS patients suffer from auditory symptoms, all should undergo pure tone audiometry testing. Obtaining both air- and bone-conduction thresholds is necessary. If the difference between air- and unmasked bone-conduction thresholds is >10 dB, bone-conduction thresholds should be masked to accurately assess the left and right ear separately. The air-bone gap (ABG) is calculated by subtracting the bone-conduction threshold from the air-conduction threshold. Many, but not all, patients with SCDS suffer from low frequency air-bone gaps (ABG) of ≥10 dB, which can be due to low or negative bone-conduction thresholds and/or elevated air-conduction thresholds. The largest ABG is typically seen at 250 Hz (12). Obviously, ABGs are not unique to SCD. They are a common finding in other otologic disorders causing conductive hearing loss, especially those with middle ear pathology (7). Therefore, further evaluation of middle ear function using tympanometry and acoustic reflexes is warranted and aids in differentiating the various causes of the ABG (7, 10). In contrast to ABG from middle ear pathology that causes abnormalities of tympanometry and/or loss of acoustic reflexes, SCD cases with ABG will exhibit normal tympanometry and preservation of acoustic reflexes. Audiometric testing alone is insufficient for diagnosis of SCD, but can be a valuable diagnostic contributor.

## Vestibular Evoked Myogenic Potentials

Tullio et al. described sound-induced activation of the vestibulo-ocular and vestibulocollic pathways in the presence of a third window (4–6). These pathways can be assessed clinically with vestibular evoked myogenic potentials (VEMP), which provide an actual physiological measurement of this phenomenon. The cervical VEMP (cVEMP) relies on the vestibulocollic reflex and assesses saccular and inferior vestibular nerve function through ipsilateral inhibition of the sternocleidomastoid muscle (13). The ocular VEMP (oVEMP) uses vestibulo-ocular projections, allowing for the assessment of utricular and superior vestibular nerve function through contralateral excitation of the inferior oblique eye muscle (14). cVEMP and oVEMP can be obtained during acoustic or vibrational stimulation of the ear while responses are recorded and averaged using surface electromyography of the contracted ipsilateral sternocleidomastoid muscle for cVEMP and contralateral inferior eye muscles during upward gaze for oVEMP (13, 14). Although various types of stimuli have been described, the most commonly used stimulus to obtain a clinical VEMP is a 500 Hz tone burst (15).

The cVEMP response consists of a first positive peak around 13 ms followed by a negative peak around 23 ms after sound stimulus onset (13). The latency of the response is dependent on the rise time of the tone burst (16). The oVEMP response consists of a first negative peak around 10 ms followed by a positive peak around 16 ms (14). The biphasic cVEMP and oVEMP responses can be evaluated using various metrics. The most clinically useful metrics are peak-to-peak amplitude and threshold. The cVEMP peak-to-peak amplitude is greatly affected by muscle contraction effort of the ipsilateral sternocleidomastoid muscle. Stronger muscle contractions correlate with greater peak-to-peak amplitudes. To allow for reliable comparison within and between patients, the peak-to-peak amplitude should be normalized for this muscle contraction effect (13, 17, 18). The oVEMP peak-to-peak amplitude is affected by gaze elevation; i.e., increased gaze elevation correlates with larger peak-to-peak amplitudes (19). Correcting for differences in gaze elevations between and within patients remains a methodologic issue that has not yet been resolved and requires further investigation. VEMPs can be obtained using varying sound frequencies and presentation levels. The VEMP threshold, i.e., the lowest sound level to elicit a response, at any stimulus frequency can provide valuable information regarding otolith function (20).

Since the presence of a third window results in activation of the vestibulo-ocular and vestibulocollic pathways as described by Tullio et al., one would expect VEMP amplitude to increase and threshold to decrease (4–6). In 1994, Colebatch et al. confirmed this prediction: A patient with the Tullio phenomenon demonstrated large cVEMP amplitudes and low cVEMP threshold (21). After Minor et al. first described SCDS, many studies confirmed that, on average, SCDS patients have larger cVEMP and oVEMP amplitudes and lower thresholds compared to healthy controls, although overlap between the SCDS and normal groups is observed (1, 20, 22–30). Until recently, the 500 Hz cVEMP threshold and 500 Hz oVEMP amplitude were found to most accurately differentiate dehiscent ears from healthy controls (20, 23).

## Enhancements of VEMP Testing in SCD

Several recent studies investigating the use of VEMP testing in SCDS patients explored new methods to improve SCD detection (12, 25, 31–33). As described earlier, there is a need for a highly specific (preferably 100%) test for SCD detection in conjunction with the highly sensitive temporal bone CT imaging.

Both the cochlea and the saccule are affected by the presence of a third window. The mechanism of SCD symptoms, shunting of acoustic energy away from the cochlea and toward the vestibular system is well-known. The resulting audiometric finding of an air-bone gap in combination with auditory symptoms such as autophony and hyperacusis is suggestive but not unique to SCD. Likewise, sound- and pressure-induced vestibular symptoms in combination with a hypersensitive VEMP response is suggestive but not unique to SCD. Multiple studies have shown that ABGs and cVEMP thresholds in SCDS patients are significantly different from healthy controls, although there is still overlap between these two groups for both metrics (1, 20, 23, 25, 26). By combining the two phenomena into one metric objective evidence is sought to demonstrate that sound energy is *both* shunted away from the cochlea and toward the vestibule, a phenomenon that really is (almost) unique to SCD or other vestibular third window disorders. Milojcic et al. investigated whether combining the ABG and cVEMP threshold would improve differentiation between SCD patients and healthy controls. ABGs and cVEMP thresholds were obtained at multiple frequencies and combining cVEMP thresholds and ABG from the same frequency, i.e., subtracting the ABG from the cVEMP threshold, increased positive predictive values at 250, 500, and 1,000 Hz (24). A later retrospective study including 142 SCD ears found that the difference in ABG between dehiscent and healthy control ears was largest at 250 Hz and showed that a calculation subtracting the 250 Hz ABG from the 500 Hz cVEMP threshold (dubbed the “Third Window Indicator”) provided better classification between SCD and age-matched healthy controls, with a sensitivity of 82% and a specificity of 100%, compared to a 46% sensitivity, and 100% specificity for the 500 Hz cVEMP threshold alone (12). A smaller prospective study, also using an age-matched healthy control group, found the Third Window Indicator (TWI) to have an 88% sensitivity and 100% specificity [Table 1; (32)]. In a group of subjects all suspected to have SCD based on symptoms, the TWI differentiated dehiscent from not dehiscent ears with a 70% sensitivity and 100% specificity (Table 2). Thus, the Third Window Indicator combines information from two sense organs (the cochlea and the saccule) that are both affected by the presence of a vestibular third window and, therefore, provides better differentiation between SCD and healthy ears compared to either of the two metrics alone.

**Table 1:** A summary of study results regarding cVEMP and oVEMP testing in a group of SCD patients vs. healthy controls.

		**Study**	***N***	**Cutoff**	**Sens (%)**	**Spec (%)**	**PPV (%)**	**NPV (%)**
*cVEMP*	500 Hz threshold (12)	Retrospective	142	<98 dB peSPL	46	100	100	35
	TWI (12)	Retrospective	142	<103 dB	82	100	100	59
	500 Hz threshold (32)	Prospective	25	<98 dB peSPL	52	100	100	79
	TWI (32)	Prospective	25	<103 dB	88	100	100	94
	2 kHz VEMPn (32)	Prospective	25	>0.67	96	100	100	98
*oVEMP*								
	500 Hz amplitude (22)	Retrospective	39	>23.5 μV	68	98	93	87
	4 kHz presence (31)	Prospective	22^∧^	n10 presence	100	100	100	100

∧ *22 patients with unilateral and 4 with bilateral SCD were included, calculations were performed with 22 ears as opposed to 30 ears. It is unclear why the remaining 8 ears were not included. For the calculation of sensitivities and specificities, temporal bone CT imaging was used as the gold standard in all studies*.

*cVEMP settings: tonebursts were generated using a Blackman gating function with a two cycle rise/fall time (4 ms at 500 Hz, 1 ms at 2 kHz) and no plateau. The 2 kHz VEMP was obtained with a 123 dB peSPL toneburst (12, 32)*.

*oVEMP settings: the 500 Hz amplitude was obtained with using toneburst generated with a Blackman gating function with a two cycle rise/fall time and a one cycle plateau at 95 dB nHL (22). The 4 kHz presence vs. absence was determined using a 7 ms long tone burst (rise/fall times unknown) at 120 dB SPL (31)*.

*cVEMP, cervical vestibular evoked myogenic potential; oVEMP, ocular vestibular evoked myogenic potential; N, number of included SCD patients; Sens, sensitivity; Spec, specificity; PPV, positive predictive value; NPV, negative predictive value; TWI, third window indicator, calculated by subtracting the 250 Hz air-bone gap from the 500 Hz cVEMP threshold; dB, decibel; peSPL, peak sound pressure level*.

**Table 2:** A summary of study results regarding cVEMP and oVEMP testing in a group of SCD patients vs. patients with SCD-like symptoms without a dehiscence (dehiscent vs. not dehiscent on CT).

		**Study**	***N***	**Cutoff**	**Sens (%)**	**Spec (%)**	**PPV (%)**	**NPV (%)**
*cVEMP*	500 Hz threshold (34)	Retrospective	25	<98 dB peSPL	42	100	100	70
	TWI (34)	Retrospective	25	<103 dB	70	100	100	80
	2 kHz VEMPn (34)	Retrospective	25	>0.67	76	100	100	85
*oVEMP*								
	500 Hz amplitude (33)	Retrospective	47	Increased^∧^	62	73	47	83
	4 kHz presence (33)	Retrospective	47	n10 presence	83	83	83	93

∧ *Increased 500 Hz oVEMP amplitude is not further defined. For the calculation of sensitivities and specificities, temporal bone CT imaging was used as the gold standard in all studies*.

*cVEMP settings: tonebursts were generated using a Blackman gating function with a two cycle rise/fall time (4 ms at 500 Hz, 1 ms at 2 kHz) and no plateau. The 2 kHz VEMP was obtained with a 123 dB peSPL toneburst (34)*.

*oVEMP settings: 500 Hz cVEMP thresholds and 4 kHz cVEMPs were obtained using tone bursts with a rise/fall time of 4 ms and no plateau. The 4 kHz cVEMP was obtained at 126 dB SPL (33)*.

*cVEMP, cervical vestibular evoked myogenic potential; oVEMP, ocular vestibular evoked myogenic potential; N, number of included SCD patients; Sens, sensitivity; Spec, specificity; PPV, positive predictive value; NPV, negative predictive value; TWI, third window indicator, calculated by subtracting the 250 Hz air-bone gap from the 500 Hz cVEMP threshold; dB, decibel; peSPL, peak sound pressure level*.

Another recent investigative interest has been the use of various stimulus frequencies to obtain VEMPs. Two studies found that cVEMP and oVEMP evoked by high frequency tone bursts provide an even better separation between SCD patients and healthy controls (31, 32). The 2 kHz normalized peak-to-peak cVEMP amplitude provided a 96% sensitivity and 100% specificity, compared to 52% sensitivity and 100% specificity of the most commonly used 500 Hz cVEMP threshold (32). The 4 kHz oVEMP (presence vs. absence) provided a 100% sensitivity and specificity, compared to 55% sensitivity, and 100% specificity of the most commonly used 500 Hz oVEMP amplitude [Table 1; (22, 31)]. Recent evaluation of these high frequency VEMPs in a clinical population, as opposed to comparison with healthy controls, found them to be highly accurate. Sensitivities, specificities, positive, and negative predictive values were 83, 93, 83, and 93%, respectively, for 4 kHz oVEMP presence vs. absence and 76, 100, 100, and 84.6%, respectively, for the 2 kHz normalized peak-to-peak cVEMP amplitude [Table 2; (33, 34)]. The 2 and 4 kHz sound stimuli are at the upper edge of the otolith organ tuning curve. Since the otolith organs are relatively insensitive to acoustic signals at these higher frequencies, vestibular activation produced by a high frequency sound stimulus is usually insufficient to provide consistent responses in normal healthy individuals. However, in the presence of a dehiscent superior semicircular canal, the otolith organ “sees” a much higher “dose” of stimulus energy due to the shunting effect of the third window, resulting in a highly reliable cVEMP (and oVEMP) response to high frequency stimuli in SCD patients.

A limitation of the studies presented in Tables 1, 2 is that CT imaging was used as a gold standard in calculating sensitivities and specificities. As described previously, CT imaging tends to overestimate the presence of the dehiscence and results in inclusion of false positives and therefore could categorize ears as dehiscent that actually do not contain a dehiscence. The alternative is to only include ears with a surgically confirmed dehiscence. This would greatly reduce the number of included ears and result in a small preselected group of patients, as many institutions use the VEMP result to determine surgical eligibility. This would result in an inflation of sensitivity and specificity. Although both methods have pros and cons, we believe that CT imaging is currently the best modality to study VEMP accuracy in detecting SCD, keeping in mind that the sensitivities and specificities may be underestimated using this method. Furthermore, we believe it is clinically relevant and a “best practice” to consider discordance of VEMP and imaging results to be a “red flag” for extra caution in consideration of the SCD diagnosis and/or surgical intervention.

## Near Dehiscence

Besides patients with dehiscent vs. normal SSC, a third group has been identified clinically: those with SCD-like symptoms and radiologic and/or surgical evidence of thin bone covering the SSC, also referred to as “near-dehiscence” (10). Symptomatology in this group can be very similar to patients with a true dehiscence, and with no significant difference in dizziness handicap (DHI) scores between the two groups (35). It is unclear why these patients have symptoms. One suggested explanation has been the potential presence of a pinpoint or “microdehiscence” that could not be observed visually (8). The first report of this phenomenon found that 500 Hz oVEMP amplitudes in 6/9 patients with a near-dehiscence to lie above the 75th percentile of healthy controls, suggesting that the VEMP may provide valuable information in this group (10).

The studies investigating the TWI and the 2 kHz cVEMP included patients with thin bone covering the SSC (i.e., near-dehiscence) as a separate group (12, 32, 34). In all studies, the ABGs, cVEMP thresholds, and normalized peak-to-peak amplitudes of the thin group were very similar to the healthy control group. No significant difference between the thin and healthy control group was found for any of these metrics (12, 32, 34). In the study investigating a clinical population in which all included patients were suspected of having SCD based on symptoms, none of the thin ears met the 2 kHz cVEMP criterion for SCD abnormality (34). This study found autophony to be the only symptom that differed among the dehiscent, thin, and non-dehiscent cohorts, being significantly more common in dehiscent patients with concordant CT and 2 kHz cVEMP evidence of dehiscence than other patients (34). In patients with discordant CT imaging and 2 kHz cVEMP results (dehiscent on CT only, but 2 k Hz cVEMP not reaching threshold for abnormality) migraine was more prevalent (34). A study investigating patients with a surgically confirmed true dehiscence vs. near-dehiscence found the 500 Hz oVEMP amplitude to be significantly higher in the true dehiscence group [*p* < 0.001; (36)]. This study did not provide sensitivities or specificities and did not include a healthy control nor clinical non-dehiscent control group (36). The study investigating the 4 kHz oVEMP in a clinical population included patients with thin bone in their group marked as negative for SCD and it is therefore unknown whether results of this group differed from controls (33). Overall, in patients with symptoms suggestive of SCD and thin bone by CT and/or intraoperative inspection, cVEMP and oVEMP tend to be normal and do not show physiologic evidence of dehiscence. The VEMP is a measure of a physiologic phenomenon of increased acoustic energy delivered to the otolith organs evoking a vestibular reflex response. The absence of an enhanced (low threshold or increased amplitude) VEMP response means energy shunting is not occurring. This seems perfectly plausible if the bone over the SCC is intact, no matter how “thin” it appears radiographically or intraoperatively. The more puzzling question is why these patients have symptoms. It seems that the thin layer of bone is sufficient to maintain normal inner ear physiology and it is unclear what underlying mechanism might account for symptoms is in these patients. This is a topic worthy of further investigation.

## Cost

Using a one-size-fits-all test battery for diagnostic evaluation of SCDS, comprising audiometric testing, cVEMP and/or oVEMP, and high-resolution CT imaging is costly. An alternative algorithmic sequential testing approach is preferable: Patients suspected of SCDS based upon symptoms and physical findings undergo comprehensive audiometry, including tympanometry and acoustic reflexes, to detect any air-bone gap, and confirm normal middle ear function. They also undergo high frequency VEMP testing. If high frequency VEMP is not available, 500 Hz cVEMP threshold can be obtained and used along with 250 Hz air-bone gap from the pure tone audiogram to calculate the Third Window Indicator. Either of these metrics, the high frequency VEMP or TWI, has extremely high diagnostic accuracy for SCD. Of all patients whose SCDS diagnosis is confirmed in this manner, only a subset will be surgical candidates: Those with significant Tullio phenomenon of sound-induced vertigo or drop attacks, those with other incapacitating vestibular symptoms, and those with severely intrusive auditory symptoms of autophony, hyperacusis, pulsatile tinnitus, and muffled hearing. Many patients with SCDS will have milder symptoms, and once fully informed of the risks vs. benefits of surgical intervention, may elect to forego surgery and live with their symptoms. Only those patients who are surgical candidates need a CT scan for anatomic assessment of their dehiscence. This approach has the dual benefits of only delivering radiation exposure to those patients with a real need and reducing overall cost by reducing the number of unneeded CT scans. Clinical application of this algorithm yielded an estimated cost reduction of 48–65% (34).

## cVEMP Methodology

VEMP testing is still an evolving field. Unlike audiometry, for example, that has been standardized worldwide, VEMP testing equipment and methodology still varies widely from site to site. The interpretation and comparison of VEMP literature is challenged by the heterogeneity of methods used. This makes it particularly challenging for clinicians to determine which settings to use and how to implement a specific VEMP protocol in their clinical practice. To provide clinicians with the available details regarding the settings of each study presented in Tables 1, 2, the figure captions include specific information regarding tone burst settings and sound levels used. Fortunately, the fact that sensitivities and specificities are similar across a number of studies investigating the same VEMP metrics using different stimulus parameters indicates that these methodologic differences may not have that great of an affect VEMP accuracy (Tables 1, 2).

The cutoff values in Tables 1, 2 could be used as an example for clinical VEMP protocols, but simply adopting these exact cutoff values into a clinical protocol without local verification may be unwise due to differences in equipment and VEMP programming. The use of newer approaches (TWI and high frequency sound stimuli) in evaluating VEMPs provide better accuracy in detecting SCD. These can be implemented with little or no modification of equipment and testing protocols currently available at many sites and we encourage physicians to implement these in their own clinics.

The studies presented in Tables 1, 2 included adult patients and used age-matched control groups. One study investigated the effect of age on cVEMP outcomes in their study group and found the expected decrease in normalized peak-to-peak amplitude and increase in threshold with age in their healthy control group. This age effect was not observed in the dehiscent group and it seems that any age effect on cVEMP outcomes is overwhelmed in the presence of a dehiscence (32). The majority of SCD patients present in their 40s and 50s and although it may not be necessary to obtain different cutoff values within the most commonly studied age range (about 25–70 years old), these cutoff values should be used with caution in “extremes of age,” i.e., those younger than 25 and older than 70 years old (12, 32).

## Discussion

Based upon the anatomy and physiology of SCD, it was predicted and subsequently confirmed that VEMP testing is a sensitive means to diagnose this anatomic condition. Over the last few years the methods have been refined to optimize both sensitivity and specificity of cVEMP and oVEMP, particularly adoption of high frequency stimuli, making it the most accurate single diagnostic test for SCD. That said, there is additional functional and anatomic information to be had from comprehensive audiometry and CT imaging.

There are several important considerations to keep in mind when using VEMP for evaluation of patients suspected of SCDS. First, it is important to prioritize a high (preferably 100%) specificity for VEMP testing in this patient group. Temporal bone CT imaging is highly sensitive for SCD (9–11). Therefore, a highly specific test adds invaluable information. In addition, SCDS treatment is surgical, making it crucial to use a test with no false positives to avoid unindicated surgery. When 100% specificity is prioritized, sensitivities of the most commonly used 500 Hz cVEMP threshold and oVEMP amplitude, only around 50%, are inadequate for clinical decision making (Tables 1, 2). Several recent developments in VEMP testing have proven to be highly sensitive and specific for SCD detection. A calculation using the 250 Hz ABG and 500 Hz cVEMP thresholds, also known as the “Third Window Indicator” (TWI), provides better differentiation between SCD patients and healthy controls compared to either test alone (12, 32, 34). High frequency cVEMP and oVEMP testing provided even higher sensitivities and specificities (31–34). The advantage of high frequency VEMP testing over TWI is that normalized cVEMP peak-to-peak amplitudes or present vs. absent n10 oVEMP can be used instead of thresholds, requiring only one recording. This reduces sound exposure and testing time.

Regarding the choice of high frequency cVEMP vs. oVEMP, a few things should be considered. At a first glance, accuracy of SCD detection appears comparable for both oVEMP and cVEMP. However, although specificities were high for both testing modalities in a clinical population (cVEMP 100% vs. oVEMP 93%), a test with no false positives is preferred for reasons described above, favoring cVEMP. It is possible that the oVEMP specificity could be improved if a certain amplitude cutoff would be used instead of a presence vs. absence criterion. However, a limitation of using an amplitude cutoff for oVEMP is the current inability to correct for differences in gaze elevation, which may limit accurate intersubject comparison and test-retest reliability.

A serious limitation of both published high frequency oVEMP studies was that some ears were excluded from analysis (31, 33). The high frequency oVEMP study using healthy subjects as the control group described 22 patients with unilateral and 4 patients with bilateral SCD (30 ears in total), while only 22 ears were included in the analysis. It is unclear which ears were excluded and why (31). The high frequency oVEMP study using a clinical control group (i.e., suspected of having SCD based on symptoms) excluded 45 ears, 73% of which were excluded because no identifiable oVEMP at any stimulus frequency or intensity could be obtained (33). It is unclear if any of the excluded ears showed a dehiscence on CT imaging (33). One possible explanation for these missing oVEMPs is subject age. The oVEMP response rate decreases with age and many healthy subjects over age 60 may not have an observable oVEMP response (37, 38).

The high frequency cVEMP studies did not exclude any ears based on cVEMP outcomes and the methods in these studies were therefore more realistic and similar to a true clinical scenario in which absent responses cannot simply be disregarded (32, 34). We do recognize that VEMP testing systems and experience with cVEMP vs. oVEMP differ between institutions. Regardless of which system or VEMP modality is used, we recommend the use of high frequency VEMP testing for SCD detection.

The cVEMP can reliably differentiate symptomatic patients with actual dehiscence from those with thin or normal bone covering the superior semicircular canal (12, 32, 34). As yet, there is no compelling explanation for clinical symptoms in those patients with thin bone on CT but normal cVEMP response. As CT imaging tends to overestimate the presence of the dehiscence, a discrepancy between CT findings and cVEMP [CT(+)/cVEMP(–)] outcomes should raise suspicion for the presence of a thin layer of bone as there is no physiologic evidence of overactivation of the vestibulocollic pathway. The only studies investigating differences in oVEMP outcomes between thin and dehiscent ears found the 500 Hz oVEMP amplitude was significantly smaller in the thin group compared to the dehiscent group, but it is unclear how much overlap, if any, existed between the two groups (36). These studies did not include patients suspected of SCD with normal CT results (10, 36).

## Conclusion

Clinical oVEMP and cVEMP testing have seen gradual evolution since they were first demonstrated to be sensitive to the presence of SCD. Based upon the high sensitivity and specificity of high frequency VEMP shown in the clinical setting, we now consider this the gold standard diagnostic screen for SCD. Combined with comprehensive audiometry, and CT if necessary for surgical planning, it is now possible to acquire a detailed physiologic, functional, and anatomic characterization of each patient's superior canal dehiscence that optimizes diagnostic accuracy while simultaneously preserving patient safety and minimizing cost.

## Author Contributions

KN and SR contributed to the conception and design, analysis, interpretation of data, and participated in drafting the article for important intellectual content. All authors contributed to the article and approved the submitted version.

## Conflict of Interest

The authors declare that the research was conducted in the absence of any commercial or financial relationships that could be construed as a potential conflict of interest.
